# HostNet: improved sequence representation in deep neural networks for virus-host prediction

**DOI:** 10.1186/s12859-023-05582-9

**Published:** 2023-12-01

**Authors:** Zhaoyan Ming, Xiangjun Chen, Shunlong Wang, Hong Liu, Zhiming Yuan, Minghui Wu, Han Xia

**Affiliations:** 1 School of Computer and Computing Science, Hangzhou City University, Hangzhou, 310015 China; 2 Polytechnic Institute, Zhejiang University, Hangzhou, 310058 China; 3 Key Laboratory of Virology and Biosafety, Wuhan Institute of Virology, Wuhan, 430071 China; 4 University of Chinese Academy of Sciences, Beijing, 100190 China; 5 Institute of Biomedicine, Shandong University of Technology, Zibo, 255000 China; 6 Hubei Jiangxia Laboratory, Wuhan, 430200 China

**Keywords:** Virus-Host Prediction, Sequence Representation, Vectorization, Deep Learning-based Sequence Modeling

## Abstract

**Background:**

The escalation of viruses over the past decade has highlighted the need to determine their respective hosts, particularly for emerging ones that pose a potential menace to the welfare of both human and animal life. Yet, the traditional means of ascertaining the host range of viruses, which involves field surveillance and laboratory experiments, is a laborious and demanding undertaking. A computational tool with the capability to reliably predict host ranges for novel viruses can provide timely responses in the prevention and control of emerging infectious diseases. The intricate nature of viral-host prediction involves issues such as data imbalance and deficiency. Therefore, developing highly accurate computational tools capable of predicting virus-host associations is a challenging and pressing demand.

**Results:**

To overcome the challenges of virus-host prediction, we present HostNet, a deep learning framework that utilizes a Transformer-CNN-BiGRU architecture and two enhanced sequence representation modules. The first module, k-mer to vector, pre-trains a background vector representation of k-mers from a broad range of virus sequences to address the issue of data deficiency. The second module, an adaptive sliding window, truncates virus sequences of various lengths to create a uniform number of informative and distinct samples for each sequence to address the issue of data imbalance. We assess HostNet's performance on a benchmark dataset of “Rabies lyssavirus” and an in-house dataset of “Flavivirus”. Our results show that HostNet surpasses the state-of-the-art deep learning-based method in host-prediction accuracies and F1 score. The enhanced sequence representation modules, significantly improve HostNet's training generalization, performance in challenging classes, and stability.

**Conclusion:**

HostNet is a promising framework for predicting virus hosts from genomic sequences, addressing challenges posed by sparse and varying-length virus sequence data. Our results demonstrate its potential as a valuable tool for virus-host prediction in various biological contexts. Virus-host prediction based on genomic sequences using deep neural networks is a promising approach to identifying their potential hosts accurately and efficiently, with significant impacts on public health, disease prevention, and vaccine development.

**Supplementary Information:**

The online version contains supplementary material available at 10.1186/s12859-023-05582-9.

## Background

Over the past decade, the number and diversity of viruses have dramatically increased. A fundamental question, particularly concerning emerging viruses that pose potential threats to human or animal health, is: who are their hosts [1, 2]? Determining the host range for these viruses through field surveillance and laboratory experiments is a time-consuming, labor-intensive, and challenging process [3, 4]. There is a growing demand for computational tools with a high degree of accuracy to predict virus-host relationships using vast amounts of genomic sequences and related datasets, which can significantly assist in identifying high-risk viruses [5].

Virus-host prediction inherently faces data imbalance and deficiency challenges, compounded with the broad viral length range. Typically, viral genome sizes vary from 0.1 to 2.5 Mb [6]. Partial sequences are available for many viruses, with the exception of complete genome sequences. Researchers have submitted a significant number of sequences to the public database for well-studied or widely researched viruses (e.g., SARS-CoV-2) [7]. However, for neglected viruses, only a few sequences have been recorded. These attributes of sequence data make it challenging for computational models to fairly and comprehensively represent each viral host category [8].

Deep neural networks for sequence modeling, such as natural language text, are powerful methods for learning the representation of text units such as words, sentences, and patterns within each category. The successful sequence models include recurrent neural networks (RNNs) [9], with typical architectures that encompass gated recurrent units (GRUs), long short-term memory (LSTM), and the Transformer [10]. These deep learning-based sequence models have been demonstrated to be more flexible and accurate in predicting significant biological traits of viruses [11–13] compared to sequence alignment-based methods, such as BLAST [14]. The state-of-the-art model VIDHOP [15] achieved the highest prediction accuracy for viral-host prediction when using an LSTM deep learning model on viral sequences. Existing models often make assumptions about the abundance and quality of training data, proposing simple representation methods such as one-hot encoding and repeat-and-cut subsequencing. However, one-hot representations are inherently sparse, memory-inefficient, high-dimensional, and equidistant between any pair of vectors. The repeat-and-cut method overlooks possible variations in subsequences. Additionally, when confronted with imbalanced virus sequence data and data deficiency, deep learning models may need help to learn adequate representations [16], resulting in severe performance deterioration [8].

To enhance the representation of viral sequences and uncover hidden virus-host patterns within them, we further exploit the continuous vector representation, originally a distributed representation for words in natural language text, and experiment with the appropriate K-mer as "words". Motivated by the success of learning general amino acid sequence representations (also known as pre-train), such as BioVec [17], seq2vec [18], and AlphaFold [19], and applying them to protein structure/function predictions [20, 21] and molecular interactions [22, 23], we propose to pre-train the vector representation of k-mers (K2V) on an extensive general viral nucleotide sequence dataset define the "sentences" by adaptively subsequencing the original viral sequences and further train the representation on a small, targeted virus-host prediction task dataset. The K2V-based sequence representations are then input into a deep neural network, which is further trained using viral sequence data labeled with host information. We name this virus-host prediction architecture "HostNet".

The experimental results have shown that HostNet outperforms the state-of-the-art method in virus-host prediction. We do not compare with models like DeepViral [3, 24] because these models employ data like protein sequences, disease phenotypes, and evolutionary signatures, which we assume are unavailable in a viral sequence-only setting. The prediction maintains stable accuracy with high data imbalance and data deficiency, where the majority class may have more than three times the number of samples than the minority class, and the minority class may have fewer than ten samples.

## Methods

### HostNet architecture

**Fig. 1:**
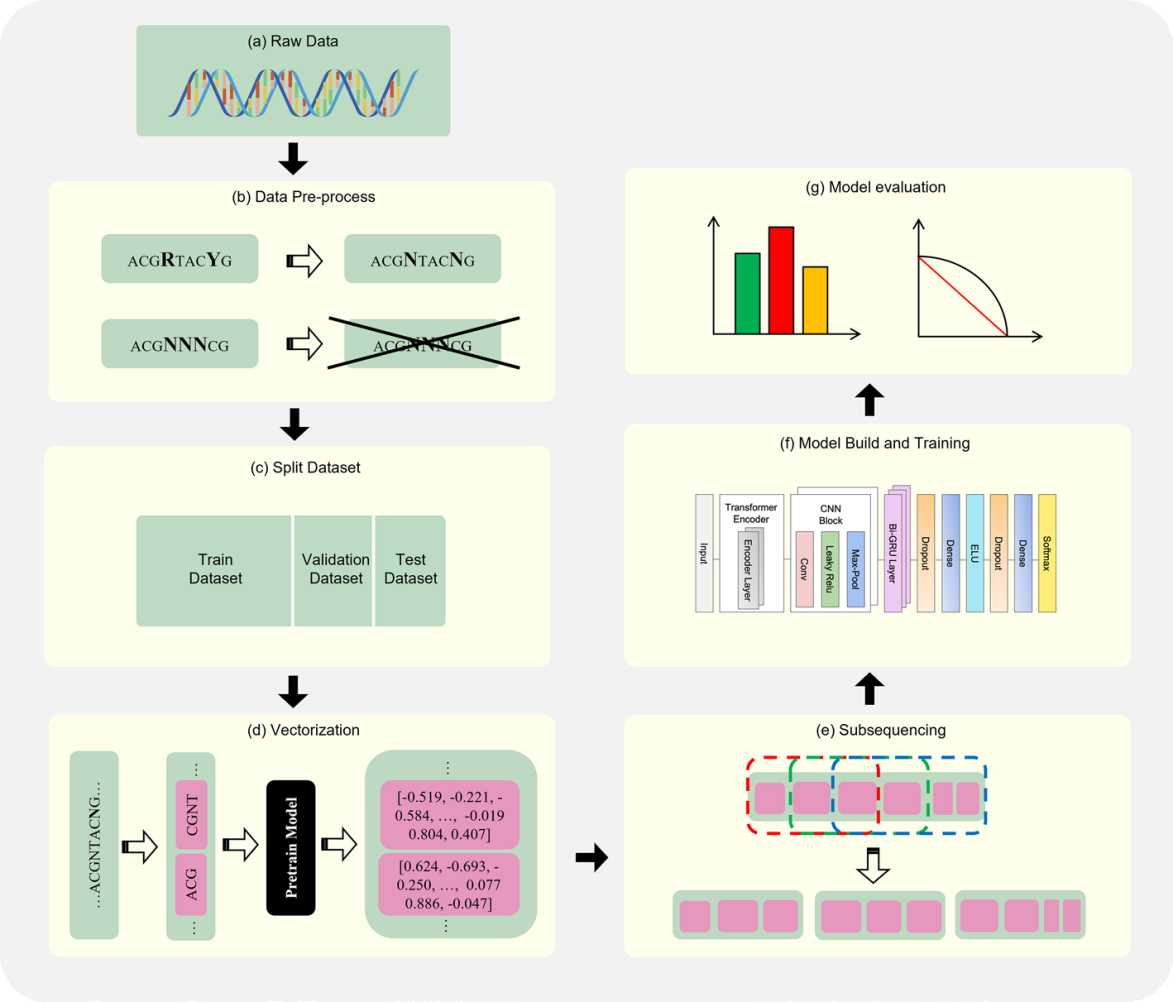
The overall architecture of HostNet. **a** The viral sequences are the original input to the system, which can be reads, contigs, or whole genomes. **b** The raw data is pre-processed by denoting single mixed bases in the sequence with N and filtering out sequences containing consecutive mixed base fragments. **c** The original data is divided into training, validation, and test datasets according to the specified proportions.** d** The genome sequence is vectorized using the K2V method and a pre-trained model. **e** The genome vector sequence is divided into subsequences by the ASW method. **f** The deep learning-based sequence analysis model contains Transformer encoder layers, convolutional layers, and BiGRU layers to capture the sequence features automatically. **g** The model's evaluation is based on metrics such as accuracy, precision, recall, and F1-score

The HostNet architecture mainly consists of a base neural network and a dedicated sequence representation component with subsequence and vectorization modules, as presented in Fig. 1.

#### HostNet’s base network: Transformer-CNN-BiGRU

**Fig. 2:**
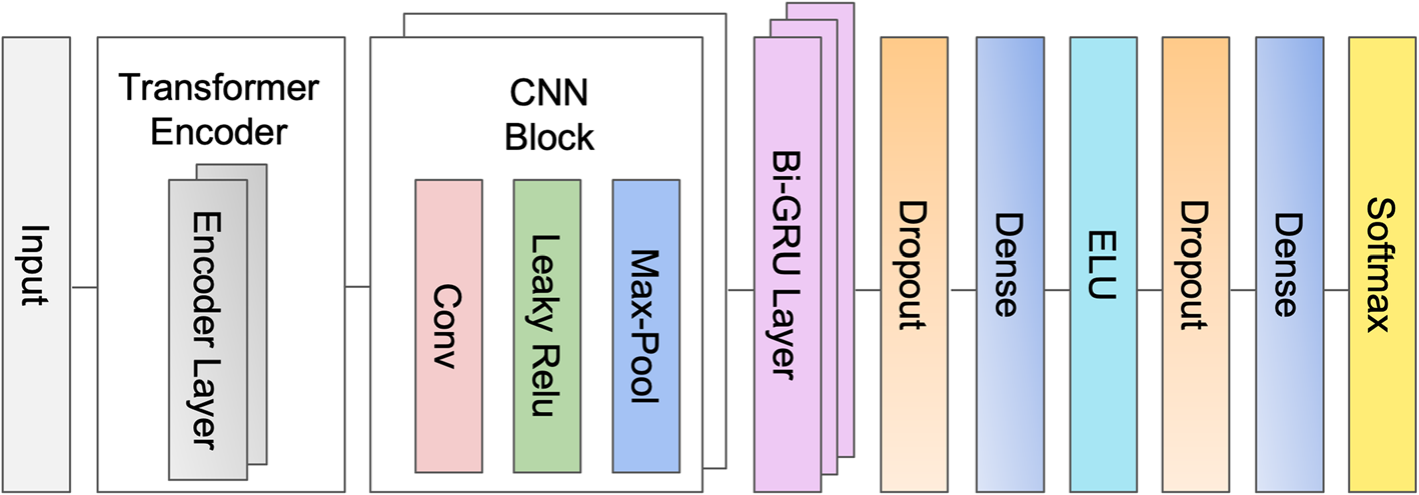
HostNet's base network. The network receives vectorized sequences as input and includes two Transformer Encoder layers, two Convolutional Neural Networks layers, three BiGRU layers, and the prediction layers

We have developed the HostNet base network, which serves as the foundational deep neural network. It consists of three distinct groups of layers: the Transformer Encoder [10] layers, Convolutional Neural Networks (CNN) layers, and the bidirectional Gated Recurrent Unit (BiGRU) layers, as depicted in Fig. 2. The inclusion of a self-attention mechanism in the Transformer enables it to capture macroscopic relationships between features within the input data, thus allowing it to adapt to the specific characteristics of the dataset at hand. Meanwhile, the CNN layers [25] are adept at fully utilizing available information within a short range and extracting the latent semantics embedded in genomic sequences. The feature extraction capabilities of CNNs also contribute to more efficient computations. Furthermore, the BiGRU layers enhance the network by integrating CNN embedding with positional information from the sequences.

The HostNet network takes a series of floating-point matrices derived from the virus sequence as its input data. This sequence undergoes a pre-processing step involving k-nucleotide embedding. Each input batch is characterized by dimensions of (1024, 250, 75). The detail information ofthe specifics of the architecture for three key modules was list as follows:

**Transformer Encoder**. The Transformer Encoder [10] is an encoder that relies on a stacked multi-head, multi-layer attention mechanism designed for sequence-to-sequence learning. It can learn internal representations and autonomously execute end-to-end text conversion. This component comprises multiple stacked encoder layers, which empowers the model to acquire and deduce global dependencies while maintaining low computational complexity.

Each Transformer Encoder consists of a stack of identical layers. Each layer is structured with a Multi-Head Attention mechanism followed by a position-wise fully connected Feed-Forward Network (FFN). In the context of this task, the primary function of the Encoder is to transform the input sequences (in this case, genomic sequences) into a sequence of continuous representations. These representations maintain the original sequence order and reveal the relationships between its elements. In our specific task, the Transformer Encoder consists of two encoder layers, each utilizing a single-head attention mechanism.

This transformed representation of the gene sequence subsequently serves as the input for the following Convolutional Neural Networks and Bidirectional Gated Recurrent Unit modules. This layered architecture enables the system to begin with one-dimensional genomic sequences and systematically abstract them into more meaningful pattern representations, which are valuable for their identification.

**CNN**. The Transformer-CNN-BiGRU architecture has two one-dimensional convolutional neural network (CNN) layers following the Transformer Encoder layers.

Convolutional Neural Networks (CNNs) [25] are commonly utilized for image classification tasks, given their ability to capture and abstract local and global patterns within an image. However, 1D-CNNs, as in this context, can also be an excellent tool for tasks involving sequential data, including genomic sequences.

In genomics, sequences are typically represented as one-dimensional data, with each element corresponding to a specific nucleotide base. Given that relevant information is often encoded in contiguous segments, the use of 1D-CNNs can be advantageous. A 1D-CNN applies a series of filters, also known as convolutional kernels, across the one-dimensional sequence. Each pass of the convolutional filter, with a specified length, over the genomic sequence involves element-wise multiplication of the filter values with the corresponding genomic sequence, followed by summation. This operation helps to identify and emphasize repeating or significant patterns within the sequence.

The result of this convolution process is a feature map that represents locations in the sequence that triggered high responses to the filter, indicating the presence of a particular genomic feature. In this case, the CNN layers perform convolution operations on the second dimension of the input data, with a convolution kernel of size 6 and stride 1. The stride dictates the step size with which the filter is moved across the sequence, and a smaller stride leads to a larger output size of the convolution. After convolution, an activation layer (Leaky Relu [26] here with a negative slope of 0.01) introduces non-linearity into the model, allowing it to learn complex patterns. Following the activation layer, a max-pooling operation is performed (with a kernel size of 2), further abstracting the representation by reducing its dimensionality and emphasizing the most prominent features.

**BiGRU**. The CNN modules are followed by Bidirectional Gated Recurrent Units [27] (BiGRU), constituted by a series of three connected Bi-GRU networks. Bidirectional Gated Recurrent Units (BiGRUs) are extensions of the original Gated Recurrent Unit (GRU) model, which is a type of recurrent neural network (RNN) used to process sequential data. BiGRUs are particularly effective for tasks where context from both past and future time steps is needed to effectively represent some information at the current time step.

In a BiGRU, two GRUs are applied to the input sequence, with one processing the sequence in a forward direction (capturing past information) and the other processing it in a backward direction (capturing future information). Each of these "directions" enables the model to accumulate temporal dependencies from the sequence's past and future, respectively. With 175 hidden nodes in this task, the network learns specific features that span over several elements in the sequence. BiGRUs design makes them well-suited for tasks where both past and future context significantly affects the interpretability of present positions. This is particularly relevant in contexts such as genomic sequences, where the representation of genes is greatly influenced by neighboring regions within the sequence.

#### Vectorization module

K2V: "K-mer to Vector" represents a more expressive vectorization method inspired by the word embedding mechanism in Natural Language Processing [28]. In bioinformatics, a "k-mer" denotes a consecutive subsequence composed of 'k' nucleotides within a more extensive genetic sequence. These subsequence fragments can encompass segments of DNA, RNA, or even protein sequences and are a universal feature across all organisms, extending beyond viruses alone. K-mers hold a prominent position in genomic analysis due to their ability to efficiently disassemble genetic information into manageable, discrete units. The flexibility of adjusting their lengths (referred to as "k" values) allows for capturing various levels of detail, rendering them highly versatile in a wide range of applications. These applications span genome assembly, error correction, sequence alignment, metagenomic classification, and many others. In our study, we treat a K-mer as a contiguous set of $k$ consecutive nucleotides within a viral sequence, mirroring the concept of a word within a sentence. K-mers collectively form a vocabulary with a size of ${4}^{k}$, considering the four types of bases present in genetic sequences. This extensive vocabulary enables the representation of diverse encoding possibilities and facilitates the capture of relationships between nucleotides within a given word. Notably, the choice of $k$ value is highly flexible, encompassing any value within the range greater than two and less than the length of the sequence itself.

In order to keep the number of types of gene sequence fragments within a reasonable range, the value range of $k$ is set to 3 ≤ k ≤ 9. We encode k-mers using one-hot encoding, input them into a neural network with a structure of two full connection layers, and adopt the Skip-gram algorithm [29] to output the front and back of each viral sequence fragment prediction results for ten k-mers.

The Skip-gram architecture is a model originally introduced in the field of Natural Language Processing (NLP) through the Word2Vec algorithm. It is employed to learn vector representations of words in a language. The fundamental idea behind Skip-gram is to predict the context, which comprises the surrounding words, given a target word. In the context of a given k-mer (the target), Skip-gram aims to predict the neighboring k-mers within a specified range, often referred to as the window size. This process closely resembles the way the model predicts contextual words in an NLP task.

After the model training completion, the parameters of the last layer of the model network are extracted, resulting in a vector of length 75 corresponding to each k-mer. For a subsequence of length ${l}_{s}$, the vectorization output is of size × ${l}_{s}$75.

One-hot representation. One-hot representation is a sequence vectorization method widely adopted in numerous studies and has demonstrated strong performance when handling raw next-generation sequencing (NGS) reads and various phenotype labels [30]. Each nucleotide and token A, T, C, G, N,—in a sequence represents a one-hot encoded vector of length six. Namely, A = [1], T = [0,0,0,0,0,1], C = [0,0,1,0,0,0], G = [0,0,0,1,0,0], N = [0,0,0,0,1,0], = − [1,0,0,0,0,0]. Subsequently, each base in the nucleotide sequence is converted into a corresponding numerical code. While the one-hot representation is straightforward and effective, it has limitations. The small vocabulary size of four (A, T, C, G) and the orthogonal relationship between the primary vectors restrict its expressiveness for representing sequences in complex tasks. Each nucleotide is independent in the one-hot representation and thus cannot fully reveal the hidden information of the viral nucleotide sequences.

#### Subsequencing module

In the context of a viral sequence s with a length $l$, the corresponding set $\{l\}$ is often observed to range from hundreds to hundreds of thousands of nucleotides. The common practice in deep learning is to utilize subsequences of a shorter length ${l}_{sub}$ compared to the original sequences. This practice prevents inefficient learning, particularly when training long neural networks on exceedingly lengthy sequences [15]. The divided $n$ subsequences are processed independently, and their predictions are subsequently aggregated to form the final result for the original sequences. In the following sections, we introduce two methods: our proposed Adaptive Sliding Window (ASW) and a state-of-the-art subsequencing method known as Repeat and Cut (RC).

In order to address the issues of unnatural concatenation and self-repetition of subsequences found in RC, we propose a partially overlapping subsequencing scheme ASW. This scheme is designed to adaptively generate a consistent number of subsequences for any given input. The underlying principles of ASW involve making optimal use of the inherent characteristics of the original genomic sequences and dividing these sequences, which may have uneven lengths, into equal subsequences without introducing unnecessary biases. ASW shares some similarities with k-mer, as k-mer utilizes a fixed stride of 1. However, ASW differs in that its stride is dynamically determined based on the original sequence's length, and the ASW window size typically spans hundreds of nucleotides, whereas a k-mer usually consists of fewer than ten nucleotides.

The ASW method is illustrated in part two of Fig. 1d. We first define the truncation stride ${l}_{stride}$ as the length between the starting positions of the two adjacent subsequences. The ASW method can flexibly adjust the size of ${l}_{stride}$ when truncating the sequences, which can be as small as one nucleotide. When we need to obtain $n$ subsequences of length ${l}_{sub}$ from a sequence of length $l,$ ${l}_{stride}$ $=$ $\frac{{l-l}_{sub}}{n-1}$. Usually, we will have ${l}_{stride}$ ≤ ${l}_{sub}$ so that the resulting subsequences completely cover the original sequence. This principle determines $n$ and${l}_{sub}$.

We need to consider the allowed longest and shortest sequences. The allowed shortest sequence is when ${l}_{stride}$ is the smallest, namely 1, whose length will be ${l}_{sub}$ $+ n-1$. The longest sequence is when ${l}_{stride}={ l}_{sub}$, whose length will be ${l}_{sub}$ * $n$. When ${l}_{sub}$ is usually preset by the model, we may adjust $n$ according to the longest sequence $\frac{{l}_{longest - {l}_{sub}}}{{l}_{sub}}$ $+$ 1 ≤ n ≤ ${l}_{longest}$  − l sub + 1. After determining n, there is a corresponding ${l}_{stride}$ for each piece of sequence in the training set, which length is ${l}_{sequence}$ and its step size is determined by ${l}_{stride}$ = $\lfloor \frac{{l}_{sequence- {l}_{sub}}}{n}\rfloor$. After the dynamically adjusted ${l}_{stride}$ is obtained, the original sequences can be truncated into a training set consisting of subsequences of ${l}_{sub}$.

As we can always set ${l}_{sub}$ smaller than ${l}_{stride}$ to allow generating partially overlapped subsequences, ASW often produces datasets larger than that obtained by the RC method. When the number of genomic sequences is small in some classes, ASW can successfully generate enough training data by using a small ${l}_{stride}$ to avoid under-fitting.

**Fig. 3:**
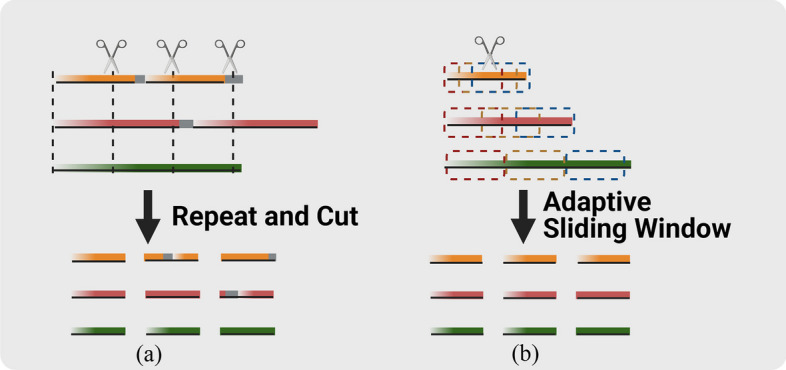
Subsequencing Methods. **a** The Repeat and Cut method repeats the sequence itself, with or without gaps. The short sequences thus reach a similar length to long sequences. The resulting sequences are cut based on the required subsequence length.** b** The Adaptive Sliding Window method uses a sliding window with a variable step to get the subsequences with the required length. ASW covers more sequence context than RC due to the coverage of subsequences

Repeat and Cut (RC). Existing methods usually truncate each sequence into $n$ multiple non-overlapping subsequences. As the original sequence length varies, this process will result in an uneven number of input units ${n}_{1}$, ${n}_{2}$, etc. Therefore, a common approach is to let the shorter sequences repeat themselves to match the longer sequences ${s}_{longest}$. Within this category, the Repeat and Cut (RC) method is widely adopted to produce the same number of subsequences from sequences of varying lengths. It self-replicates the original sequence into ${s}_{extend}$ with a shorter length into a comparable length as the longest sequence. After the extension, all sequences are cut into non-overlapping chunks of a few hundred nucleotides long. The result is that all original sequences produce the same number ${n}_{uniform}$ of subsequences of a uniform size. RC is simple and eff ective, representing the state-of-art method in the previous works. We show the comparison between ASW and RC in Fig. 3.

### Data processing and training details

Data processing involves three key steps: cleaning, deduplication, and splitting. In the cleaning phase, we employ duplicate reduction based on sequence similarity and the sequence's GenBank ID. Additionally, unconventional bases—those other than A, T, C, G, and N (representing an unknown base)—are substituted with the base N. If base N occurs consecutively more than 20 times within a sequence or if the count of N exceeds 5% of the total base count, we consider it low-quality and discard the sequence. In cases where uncertain bases like N are encountered, in K2V pretraining, we randomly replace N with one of ATCG. Subsequently, we partition the dataset into three disjoint sets for training, validation, and testing. The training set is used for fitting the virus-host prediction model; the validation set assists in fine-tuning the parameters of our proposed methods; and the test set is held in reserve to assess the model's effectiveness on previously unseen data.

We implemented a weighted Cross-Entropy loss function for training our network, which is widely adopted for classification tasks. The loss function is adjusted by class-associated weights to balance the uneven distribution of data across classes. The basic principle is to adjust the training loss of the majority class and minority class so that each class contributes evenly to the overall loss and model updating.

Minority classes are typically defined as those containing fewer than 10% of the samples compared to the majority classes. For instance, in the Rabies Lyssavirus dataset, "*Artibeus lituratus* (90)", "*Cerdocyon tous* (86)", and "*Lasiurus borealis* (96)" are considered as minority classes. The minority classes generally present as challenges for the computation models. However, it does not mean the absolute size of the minority class is small. For example, in the Flavivirus dataset, the least represented class "*Ixodes (373)*" consists of hundreds of examples. The majority classes usually have more than a few hundred samples. For examples "*Canis lupus* (5121)" and "*Bos taurus *(2280)" in the Rabies Lyssavirus dataset, and "*Culex *(2424)" and "*Aedes *(6829)" in Flavivirus dataset.

During the back-propagation of the model training process, the loss of each sample in the minority class is up-weighted, while the loss of each sample in the majority class is down-weighted. To achieve the up or down-weighting effect, we designed weighting factor ${w}_{i}$ for the loss of samples from class $i$ ∈ (1, …, $i$, …, N), where N is the total number of classes. Denoting the number of training data of class $i$ as ${n}_{i}$, we defined ${w}_{i}$ as $\frac{\sum {n}_{i}}{{n}_{i}}$. During training, we multiplied ${w}_{i}$ by each sample loss during the back-propagation process to balance the classes. This loss reweighting approach has been proven to achieve the oversampling effect [31] without increasing the computational space and time.

We trained the HostNet model using the Adam optimizer with a learning rate set to 0.001 and a batch size of 64. The employed loss function was cross-entropy, and we applied early stopping to mitigate over fitting. Our models were constructed using Python version 3.8.5, torch 1.11.0 + cu113, with the Nvidia GeForce RTX 3090 serving as the training platform.

## Materials and results

In this section, the datasets were curated and the experiments were carried out to assess the essential characteristics of the proposed balancing methods. Our results will be presented based on the test set, and the best models will be chosen based on the independent validation set.

### The Datasets

To benchmark the proposed method HostNet, a vertebrate host dataset Rabies lyssavirus, and an invertebrate host dataset Flavivirus are adopted. We have also constructed a pre-training dataset Vir61 for the K2V representation learning. The specifics of these datasets are provided below.

#### Rabies lyssavirus

We adopt a vertebrate host prediction dataset of the virus species Rabies lyssavirus from VIPHOD [15] to benchmark the balancing methods. “Rabies lyssavirus” is a typical virus that can infect humans and animals. The final dataset consists of 11,685 genomic sequences composed of 17 known host species. We used 90% of the dataset for training and 10% for testing and validating.

#### Flavivirus

For invertebrate host prediction, we curated a nucleotide sequence dataset named Flavivirus (https://figshare.com/ndownloader/files/43234947) comprising the viruses belong to the genus of *Flavivirus* from Genbank with the information of their invertebrate host lables [32]. The dataset encompasses 9,626 sequences representing 80 flaviviruses categorized according to their three primary invertebrate hosts: two mosquito genera, *Culex* and *Aedes*, and one tick genus, *Ixodes*. Within this dataset, 64 viruses were found to have mosquitos as their hosts, and 16 viruses had *Ixodes* as their hosts. The dataset included 2,424 sequences for *Culex*, 6,829 sequences for *Aedes*, and 373 for *Ixodes*. For the Flavivirus dataset, we allocated 80% of the data for training purposes and reserved the remaining 20% for testing.

#### Vir61

To facilitate the training of the k-mer to vector representation model, we built a large-scale, wide-ranging, and comprehensive viral nucleotide sequence dataset named Vir61 (https://figshare.com/ndownloader/files/43234938) [32]. This dataset draws its virus nucleotide sequence sources from GenBank. Within the Vir61 dataset, there are a total of 103,466 viral genomes spanning 1,377 viruses, representing a diverse array of 60 viral families and one unclassified set, including but not limited to *Rhabdoviridae*, *Togaviridae*, *Ascoviridae*, and *Flaviviridae*. For training the K2V model on the Vir61 dataset, we conducted 50 epochs with a learning rate set at 0.001 and a batch size of 256.

**Fig. 4:**
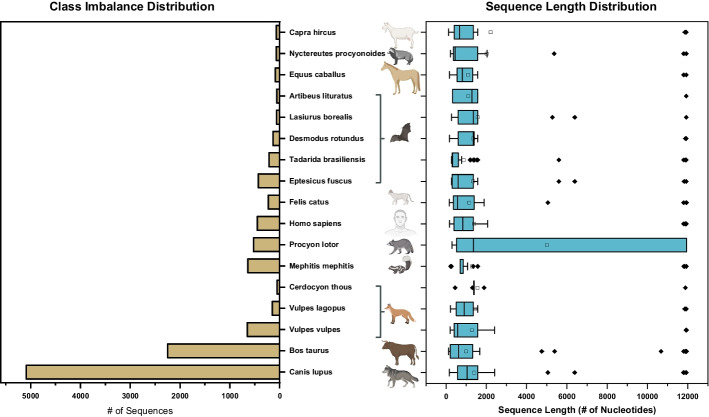
Distribution of sequences in the Rabies lyssavirus dataset. The left panel shows the class distribution, while the right panel shows the sequence length distribution. The compound effect of these two aspects of distribution biases creates an overall imbalance in the dataset when training a deep learning-based viral sequence model

The statistics for the three datasets can be found in Additional file 1: Tables S1, S2, and S3. To illustrate the characteristics of a typical viral host prediction dataset, we will use Rabies lyssavirus as an example. Figure 4 shows a long-tail distribution of the class sizes within the benchmark dataset. Notably, more than 85% of the categories contain fewer than 1000 viral sequences, and 35% have less than 100. To quantify the class distribution imbalance, we define the class imbalance ratio as the number of samples in the majority classes divided by the number of samples in the minority class [16]. In the benchmark dataset, the majority class, "*Canis lupus*", contains over 5000 viral sequences, while the minority class, "*Cerdocyon thous*", contains only 50 sequences. This results in a class imbalance ratio of approximately 100.

In addition to the class-level imbalance, there is also a noticeable imbalance at the sequence level. As shown in Fig. 4, the typical range of sequence lengths falls between 150 and 1000 nucleotides, with outliers reaching up to 10,000 nucleotides in length. Given the varying lengths of sequences within each host class, and in combination with the class distribution imbalance, the distribution of input data units (subsequences) can become significantly more skewed. The dataset contains sequences ranging from the longest at 11,939 nucleotides to the shortest at 117 nucleotides. This results in a sequence length imbalance ratio of approximately 75.

### Evaluation metrics

To evaluate the prediction performance of the methods, we segmented the test sequences into fixed-length subsequences without extending the original sequences. We used the following metrics to evaluate the prediction performance:

• Accuracy Standard: measures the proportion of correctly predicted subsequences.

• Accuracy Aggregated: measures the proportion of correctly predicted sequences. The prediction for each sequence is aggregated by calculating the mean activation score per class on all subsequences and predicting the class with the highest mean activation.

• F1-score: a harmonic mean of Precision and Recall defined in Eq. 1. It is a valuable metric for evaluating methods when the category distribution of data is imbalanced.

$$Precision = \frac{True\;Positive}{{True \;Positive + False\; Positive}}$$

1 $$Recall = \frac{True\;Positive}{{True\;Positive + False\;Negative}}$$

$$F1 - score = \frac{1}{1/Precision + 1/Recall}$$

### HostNet performance benchmarking

In order to benchmark the performance of HostNet and its key representation components, ASW and K2V, we implemented several variants of the method, including HostNet with and without ASW and K2V, as well as a state-of-the-art method called VIDHOP [15]. VIDHOP consists of several variants, including two backbones (CNN + BiLSTM and BiLSTM) and four sequence segmentation and balance methods (normal repeat, normal repeat with gaps, random repeat, and random repeat gaps). VIDHOP adopts one-hot vectorization and does not use pre-trained models. In this work, we report the best results among the VIDHOP variants.

We utilized BlastN to establish a comparative baseline for assessing HostNet's performance. Our approach involved querying the viral sequences against a database of host organisms. After obtaining the similarity scores, we inferred the probable host by identifying the highest similarities between the viral and host sequences. The host with the most similar sequence was predicted as the probable host.

**Fig. 5:**
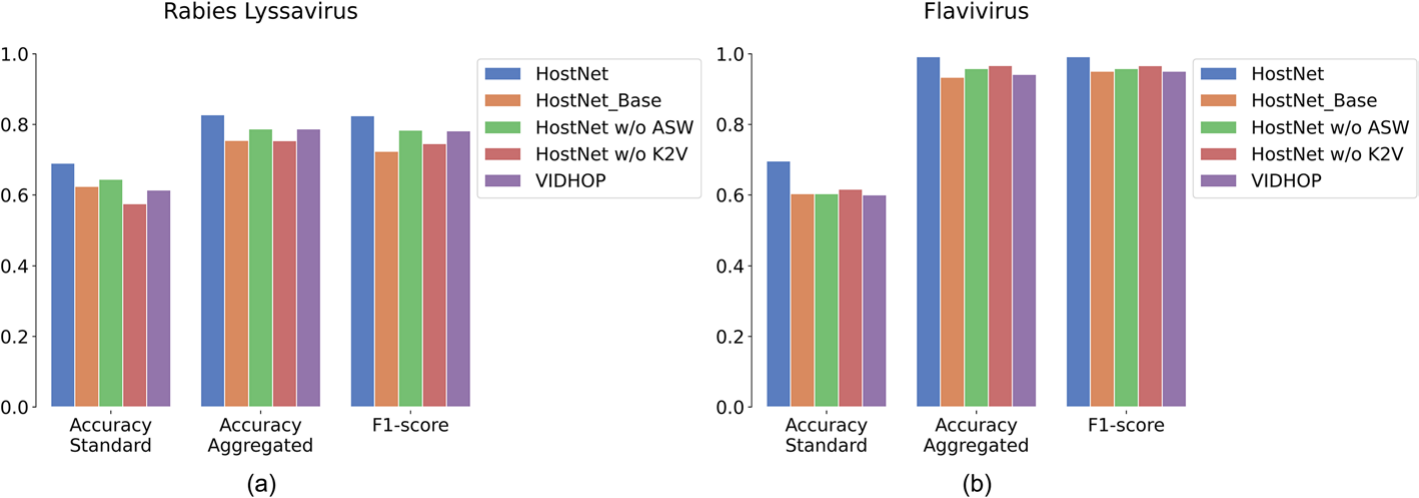
HostNet benchmarked against state-of-the-art and HostNet variants. The performance was evaluated based on the accuracy (standard and aggregated) and F1-score. The grey bars represent HostNet; the left striped bars represent HostNet Base; the right striped bars represent HostNet without ASW; the cross striped bars represent HostNt without K2V; and the sparsely dotted bars represent VIDHOP. **a** The viral host prediction results on the Rabieslyssavirus test set. **b** The viral host prediction results on the Flavivirus test set

The comprehensive comparison of methods on the two datasets is presented in Fig. 5. The results indicate that HostNet consistently surpasses the other methods in terms of accuracy (standard and aggregated) and F1-score on both datasets, with a significant margin over VIDHOP.

Furthermore, we observed that removing either ASW or K2V from HostNet results in a considerable drop in performance, indicating that both components contribute significantly to the HostNet framework.

Comparing the performances of the two datasets, we found that while the accuracy standard is similar at around 0.6, the Flavivirus dataset has higher accuracy aggregation and F1-score. This suggests that HostNet provides consistent base predictions on datasets with different numbers of classes. The key difference between the two datasets is that our in-house Flavivirus dataset consists primarily of complete sequences, while the Rabies lyssavirus dataset may contain fragments. Therefore, complete sequences may result in better overall predictions when aggregation is performed.

### HostNet is stable with increasing imbalance ratios (checked)

Although the benchmark dataset used in this study is naturally imbalanced, with the minority class containing only 86 samples, it may still over-represent data abundance in general viral host prediction tasks, where a host may have fewer than ten virus samples. Real-world scenarios often involve such class imbalance, where one class may only have a few samples while others may have hundreds or thousands.

**Fig. 6:**
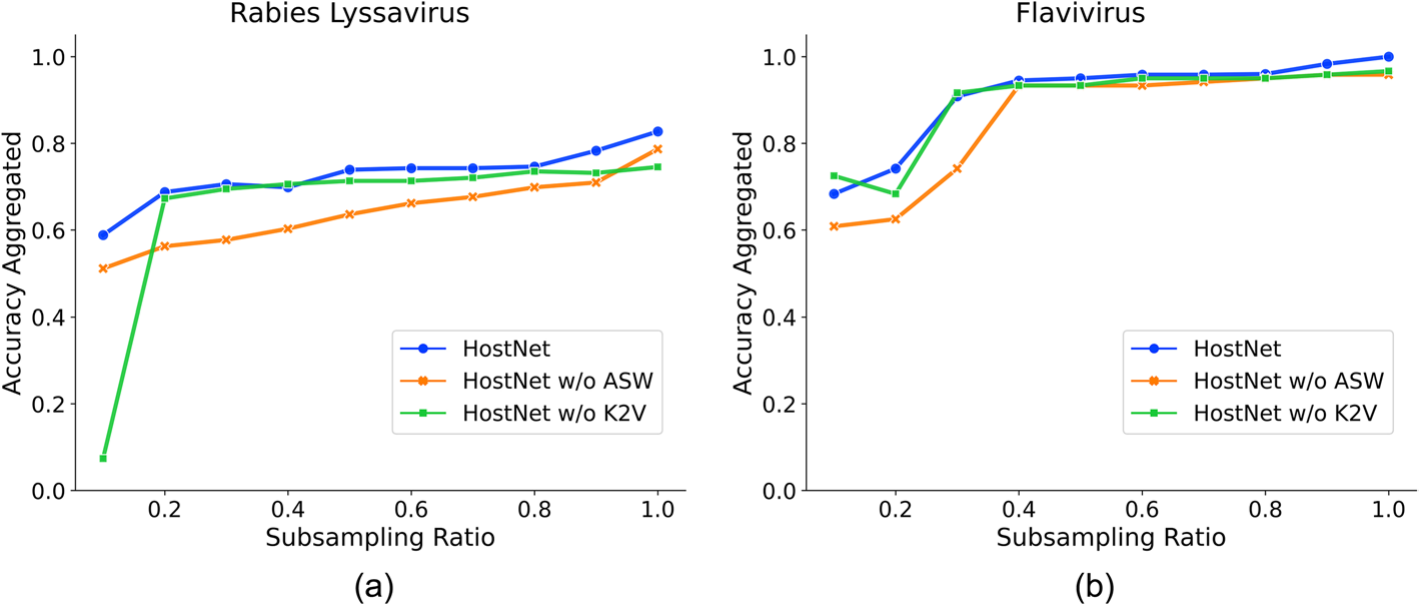
The learning curves for showing the convergence of HostNet, HostNet without ASW, and HostNet without K2V during training on the training and validation datasets. **a** Training and validation accuracy (standard) during training on the Rabies lyssavirus dataset. **b** Training and validation accuracy (standard) during training on the Flavivirus dataset

We formulated training data by gradually reducing the number of training samples for the host classes with the least data to evaluate how the balancing methods respond to increasing imbalance ratios. Specifically, we decreased the training samples for classes that had fewer than 100 sequences, which included "*Artibeus lituratus* (90)", "*Cerdocyon tous* (86)", and "*Lasiurus borealis* (96)" in the Rabies Lyssavirus dataset, as well as the most minor class, "*Ixodes* (373)", in the Flavivirus dataset. We generated ten training sets by randomly subsampling from 10% up to 100% of sequences from these three underrepresented classes while keeping the other classes unchanged. The prediction accuracy under these imbalanced datasets is illustrated in Fig. 6.

From Fig. 6, we can see that HostNet exhibits stable performance on the two datasets across all subsampling ratios. Even at the 10% subsampling ratio, both datasets maintain a 0.6 accuracy. This finding is particularly noteworthy given that the least represented class in the Rabies lyssavirus dataset has less than ten training samples. Therefore, our study establishes HostNet's capability to work effectively on highly imbalanced datasets. Moreover, our analysis shows that the accuracies are almost stable, hovering around the original dataset accuracy level of 100%, in the range from 40 to 90%. This result indicates that HostNet is resilient to a range of imbalance levels.

By replacing the vectorization method K2V with one-hot encoding (HostNet w/o K2V) and the subsequencing method ASW with repeat and cut (RC) (HostNet w/o ASW), we observed a significant deterioration in performance, particularly at low subsampling ratios (high imbalance ratios). This finding demonstrates that both K2V and ASW contribute to the imbalance resilience of the HostNet framework.

### HostNet captures hidden patterns in predicting challenging viral hosts

**Fig. 7:**
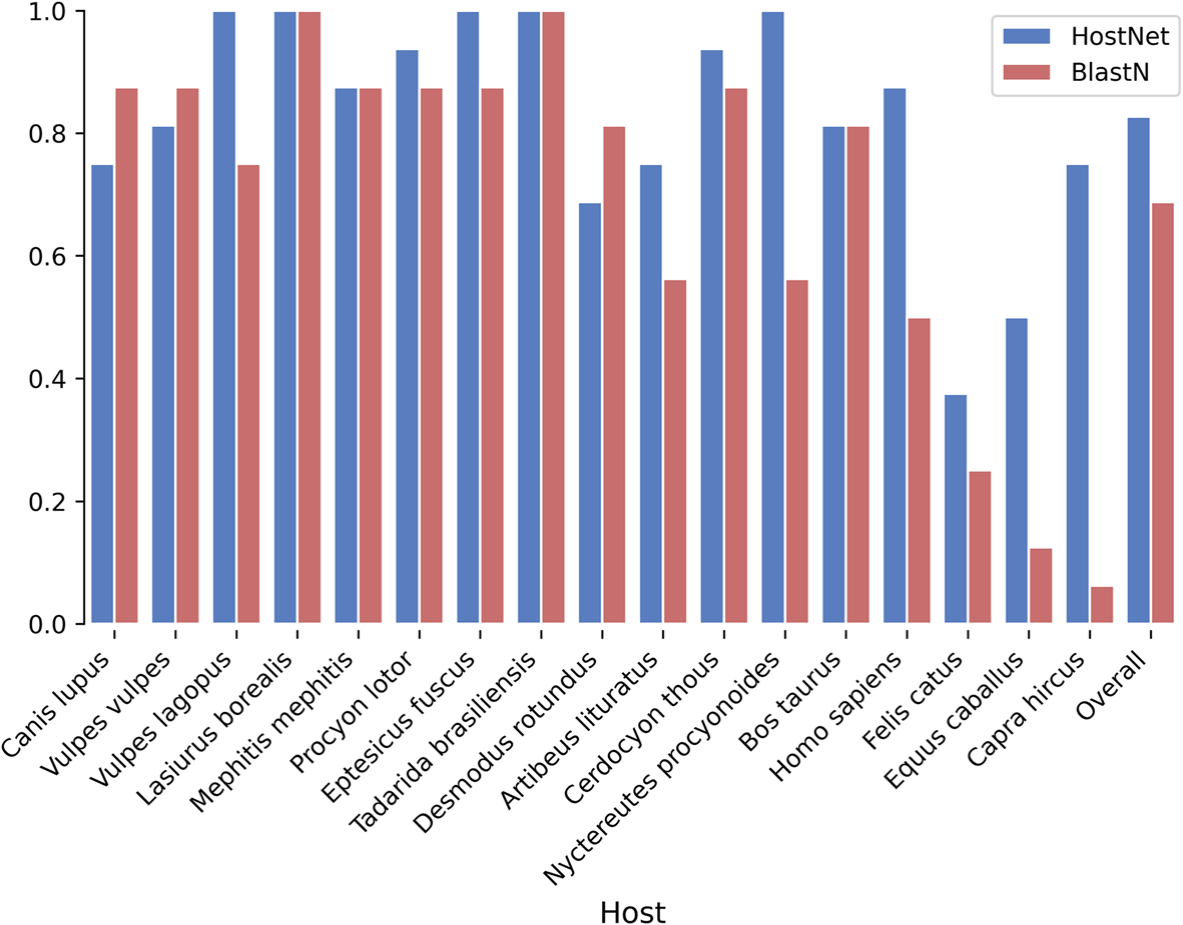
The per host prediction performance by HostNet and BlastN on Rabies lyssavirus dataset. The densely dotted bars represent HsotNet performance in Accuracy Standard; the sparsely dotted bars represent BlastN performances in Accuracy Standard

To quantify the surface sequence similarity among viruses within a host class, we adopt BlastN [33]. When BlastN [33] fails to predict viral hosts based on sequence alignment, it indicates insufficient sequence similarity to capture the characteristics of the host class. As an example, consider the hosts "*Equus caballus*" and "*Capra hircus*" as shown in Fig. 7. BlastN [33] yields an accuracy below 0.1 due to the low sequence similarity among viral sequences within each host. However, HostNet provides reasonably accurate predictions, surpassing 0.5. This suggests that HostNet is capable of identifying hidden patterns in the sequences that escape detection by the sequence alignment method.

**Fig. 8:**
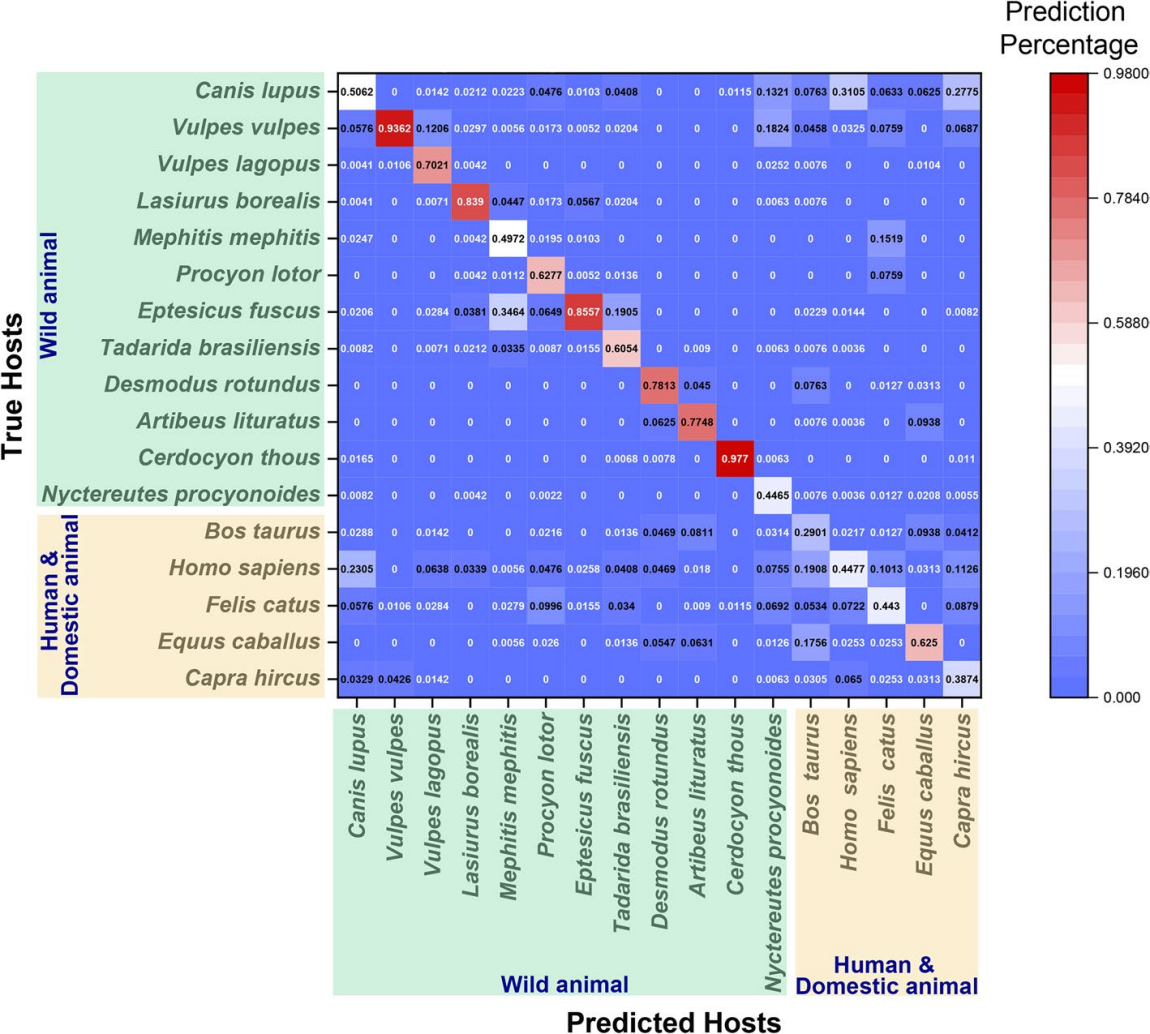
The viral host prediction confusion matrix. The accuracy (standard) is reported based on the uniformed length subsequences. The hosts in the Wild animal and Human&Domestic animal categories are highlighted separately. It shows that the Human&Domestic animal category is more challenging

From the confusion matrix in Fig. 8, we observe that the virus-host prediction performance in the Human&Domestic animal category is less accurate compared to the Wild animal category. The predictions are more likely to be confused in the Human&Domestic animal category than in the Wild animal category. This suggests that predicting hosts that belong to the Human&Domestic categories is more challenging than predicting those in the Wild animal category. This may be due to the fact that the viruses infecting domesticated animals or humans originated from those infecting wild animals and thus exhibited higher diversity in genetic composition.

To further compare the per-host accuracy (standard) performance on the Rabies lyssavirus dataset, we examine the BlastN [33] results in Fig. 7. We observe that some host categories have viruses that share high sequence similarity, while others have low sequence similarity. Despite this variation, HostNet provides reasonably high performance across all host categories regardless of viral sequence similarity. BlastN [33], on the other hand, does not perform well on the five domesticated animal or human hosts, indicating the low sequence similarity among virus sequences within each host. Nevertheless, HostNet predictions for these hosts maintain a similar level of accuracy as for the wild animal hosts, suggesting that HostNet captures deeper patterns of the host category than BlastN [33].

### ASW and K2V enhance generalization in HostNet

**Fig. 9:**
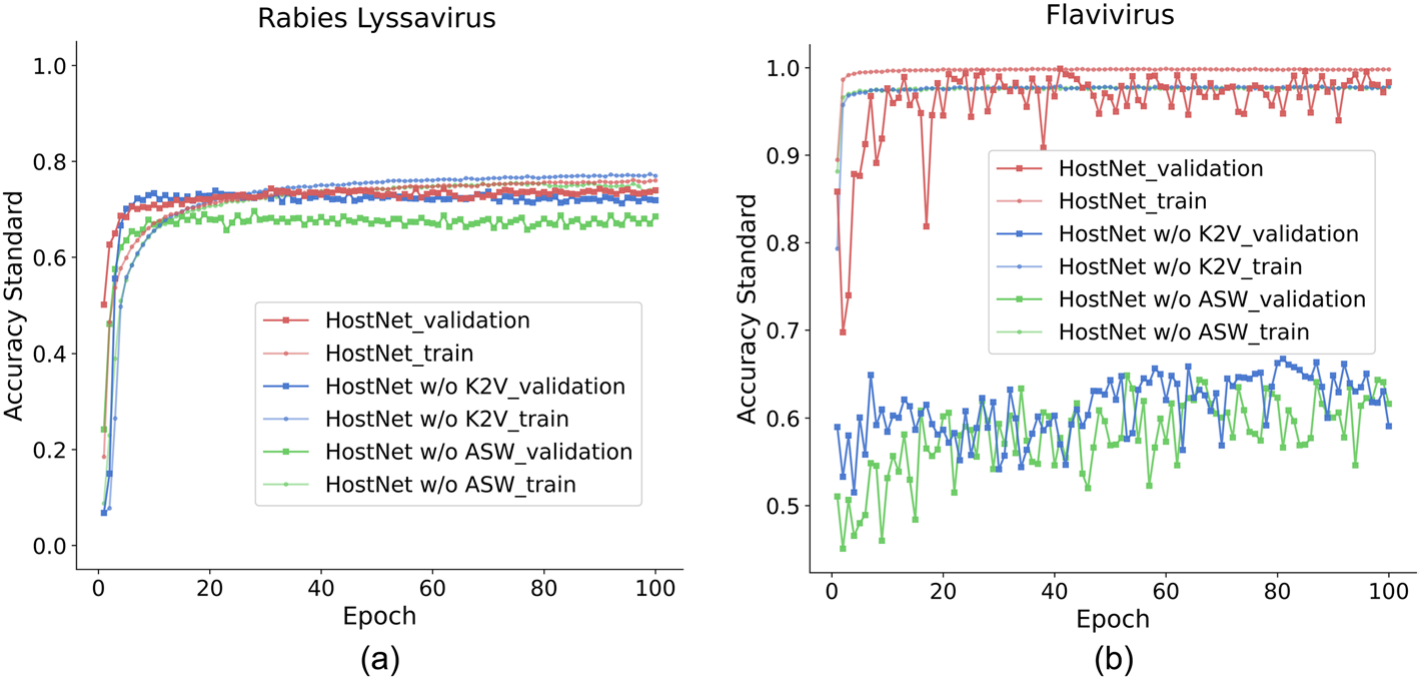
The learning curves for showing the convergence of HostNet, HostNet without ASW, and HostNet without K2V during training on the training and validation datasets. **a** Training and validation accuracy (standard) during training on the Rabies lyssavirus dataset.** b** Training and validation accuracy (standard) during training on the Flavivirus dataset. The dotted line with empty green triangles represents the learning curve of HostNet's Accuracy Standard on the training set. The solid line with solid green triangles represents the learning curve of HostNet's Accuracy Standard on the validation set. The dotted line with empty red squares represents the learning curve of HostNet without ASW’s Accuracy Standard on the training set. The solid line with solid red squares represents the learning curve of HostNet without ASW's Accuracy Standard on the validation set. The dotted line with empty blue circles represents the learning curve of HostNet without K2V's Accuracy Standard on the training set. The solid line with solid blue circle represents the learning curve of HostNet without K2V's Accuracy Standard on the validation set

We evaluated HostNet and its components to assess their generalization abilities by comparing their potential for overfitting. An overfitting model demonstrates low loss during training but performs poorly when predicting new data. Figure 9 displays the standard training and validation accuracy during model training. As training progresses, the accuracies tend to converge, and we made the following observations regarding the learning curves generated during this process.

Firstly, HostNet displays the smallest gap between the training and validation accuracy, indicating its highest generalization ability among the three. HostNet's validation accuracies are the highest on the two datasets, although its training accuracies are not consistently the highest. HostNet without K2V demonstrates the highest training accuracy on the Rabies lyssavirus dataset but does not make the most accurate prediction on the validation data. This observation suggests that K2V is a contributing component to HostNet’s generalization ability. Pretraining on an external large dataset provides a generic base representation, which is further refined with the task’s training data. A similar observation is made on ASW, which captures subsequence information more effectively than RC and balances the number of generated subsequences.

Secondly, HostNet's validation curves are the smoothest, with the smallest fluctuation after approximately 50 epochs. Conversely, although HostNet without ASW and HostNet without K2V have smooth training curves, their validation curves fluctuate significantly from the beginning of the training process until the late stage. This result demonstrates that HostNet’s generalization is robust due to the improvement brought by ASW and K2V representation.

In summary, the evaluations reveal that HostNet with ASW and K2V enhances the generalization ability of the model, which is reflected in the smooth validation curves and the smallest gap between training and validation accuracy.

### Optimizing HostNet representation with pretrain parameter K

**Fig. 10:**
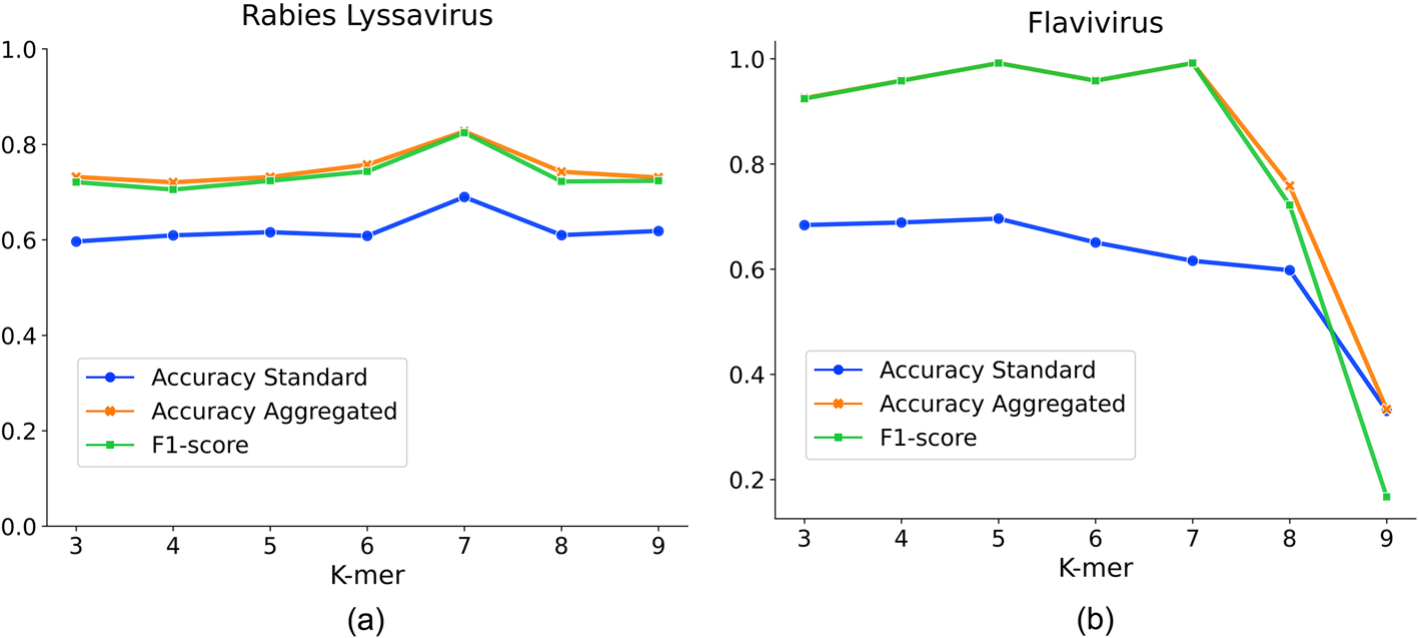
The effect of k-mer size k for pertaining the sequence representation. The three lines show three evaluation metrics: Accuracy Standard, Accuracy Aggregated, and F1-score. **a** Validation performance of different k on the Rabies lyssavirus dataset. **b** Validation performance of different k on the Flavivirus dataset

Our experiments have demonstrated that the K2V representation, pre-trained on an external dataset, is a critical component of the HostNet framework. The k-mer size k is a crucial hyper-parameter in the HostNet optimization process. To determine the optimal k for the experiment, we experimented with a range of k on the two datasets and presented our findings in Fig. 10.

Based on the results shown in Fig. 10, we have set k to 7 for the Rabies lyssavirus dataset and 5 for the Flavivirus dataset. Figure 10a demonstrates that the optimal k for the Rabies lyssavirus dataset is 7, whereas Fig. 10b peaks at k = 5 for the Flavivirus dataset. Additionally, we observe that the curves remain relatively stable within the k = 3 to k = 7 range, indicating that the choice of k is not hyper-sensitive to HostNet performance over a considerable range. Thus, identifying a suitable k for HostNet when applied to a new dataset is not challenging.

We note that performance drops when k is set to 8 or 9 on both datasets. We conjecture that this may be due to the deterioration of pre-training quality as a result of the larger k value. Larger k entails a larger vocabulary size and more complex patterns to be captured over a longer range, which generally requires a larger training dataset. Based on the results of our experiments, we conclude that k = 3 to 7 could be a suitable range for our pre-training dataset to generate high-quality base representations.

## Discussion

We have proposed HostNet, a deep learning-based method for virus-host prediction, which incorporates improved sequence representation via ASW subsequencing and K2V vectorization components.

HostNet is applicable to future virus-host prediction tasks due to the persistence of issues faced in this work, including data deficiency, data imbalance, and sequence length distribution. These issues are fundamental due to the random nature of viral sequence data collection, which is affected by multiple social and environmental factors. HostNet, including ASW, K2V, and the sequence modeling neural network, remains effective for these tasks.

The ASW approach is suitable for most cases involving sequence length imbalance. It blends well with vectorization methods and sequence modeling architectures, making it superior to the state-of-the-art repeat and cut method. It requires replicating genomic sequences with shorter lengths multiple times, introducing more gaps, and affecting the training set data quality. ASW has an additional advantage as each subsequence’s position is determined, making it suitable for position-sensitive subsequence analysis.

K2V, or the pre-trained model, is reusable and applicable for representing viral sequences in host prediction tasks. The generic representation acquired from a vast viral sequence dataset can compensate for the data deficiencies in certain viral host categories, while a finely-tuned representation based on a smaller dataset will be sufficient for others. To further improve performance, increasing the closeness and comprehensiveness of the pre-train dataset related to the application tasks is essential.

The highly adaptable nature of HostNet's architecture makes it capable of implementing advancements and improvements as they become available. Future updates could occur in various layers of the architecture, depending on the progress in the field of deep learning. For instance, improvements to HostNet could come from advancements in transformer architectures. Currently, transformer architectures are rapidly evolving, with novel designs like Vision Transformers emerging. Integration of such advancements could potentially enhance HostNet's capacity for processing genomic sequences.

Similarly, improvements in recurrent layers, such as Gated Recurrent Unit (GRU) or Long Short-Term Memory (LSTM), could offer promising updates. New variants that facilitate more efficient learning of temporal dependencies or address the issue of vanishing gradients could be seamlessly integrated into the HostNet model. Furthermore, updates to the convolutional layers are critical. Given the emergence of novel convolutional techniques, such as dilated or separable convolutions, their incorporation could enhance HostNet's sequence processing capabilities.

HostNet's improved sequence representation broadly applies to capturing biological sequence patterns. In this work, our evaluation is based on RNA viruses. However, when considering DNA viruses, host prediction can significantly differ due to the difference in their replication strategies and genetic makeup. DNA viruses typically establish more stable interactions with their hosts, while RNA viruses exhibit higher mutation rates, which may alter host predictions. Nevertheless, the robustness of HostNet could tolerate these challenges. Its unique pre-trained k-mer to vector method enables the effective representation of various types of viral genomic sequences and handles the high mutation rates characteristic of RNA viruses. Another possible area of application is predicting retrovirus hosts, which introduces an additional layer of complexity as these viruses integrate their DNA into the host genome. Fortunately, the ASW technique offers high-throughput data processing capabilities, which could be valuable in processing such complex scenarios.

There are challenges and limitaitons that remain to be solved under the representation-enhanced deep learning framework. HostNet, as a representative of deep learning-based genomic sequence modeling methods, has shown higher accuracy and efficiency at the testing time. However, the training of any deep learning model is time and resource-expensive. It requires more data to train the models and computational resources like GPUs, which consume substantial energies for complex computations. HostNet, like other deep learning models, utility is bounded by the data categories that are defined by the training data. The model training process may not be robust, as evidenced by the fact that initialization and hyperparameter choices may affect the performance significantly. The tuning of the hyperparameters may entail many iterations of training to reach the optimal models, which is a process that may not be theoretically determined and relies on empirical trial and error. Furthermore, the model's performance was tested on two datasets, evaluating the model on a wider variety of datasets should be done in the next step to further validate its robustness. Biologically, the deep learning model’s ability to infer host-virus associations from genomic sequences is a complex task that may not capture all aspects of the viral host range, such as ecological, immunological, and physiological factors that also play crucial roles in determining host susceptibility to viruses. We haven't come to any concrete conclusion on our exploration of the model's interpretability, it was one of the limitations of this study.

Some viruses may have multiple hosts, and some have a complex co-evolutionary history with their hosts. Genome-wide nucleotide substitutions or deletions/insertions attributed to viral genome diversity are responsible for the change in the host shift of the virus [34]. SARS-CoV and SARS-CoV-2, for example, are presumed to have originated in bats and infected humans with spillover to domestic and wild animals [35]. Most existing training data contains genomic sequences uniquely associated with a single host each, which is unsuitable for making accurate predictions for those with multiple hosts. Our experiments also reveal that viruses that infect domesticated animals and humans are more complex than those infecting wild animals, making it challenging for the model to make accurate predictions. The host genomic sequence can be an additional source of information for broadening viral host prediction. It can benefit the multiple host prediction, as well as the virus-host co-evolution analysis. In a possible direction, a virus-host pair can be evaluated for their possible relation.

To sum up, HostNet represents an effective approach for viral-host prediction tasks, offering improved sequence representation and a generic network architecture that is amenable to future updates. Nonetheless, further research is needed to address the challenges associated with multiple hosts, complex co-evolutionary histories, and a wide variety of host categorizations and interaction patterns.

## Conclusion

In conclusion, we have proposed a deep learning-based method, HostNet, for predicting virus hosts based on genomic sequences. Our proposed framework addresses the challenges posed by sparse and varying-length virus sequence data through two enhanced sequence representation modules: K2V and ASW. HostNet significantly outperforms state-of-the-art deep learning-based host prediction methods on the Rabies lyssavirus and the Flavivirus datasets. The proposed representation scheme improves the learning and generalization capabilities of the model, making accurate predictions with naturally imbalanced datasets and sequences of varying lengths. The results demonstrate the potential of HostNet as a valuable tool for predicting potential virus hosts in various biological contexts. Our work contributes to advancing the field of viral host prediction, potentially providing timely responses in the prevention and control of emerging infectious diseases and further empowering drug discovery and vaccine development. Recent emerging large language models (LLMs) like GPTs are showing more advanced power in understanding and generating languages. The genomic sequences as the language of life may also benefit from the advances in biological LLMs in the future.

### Supplementary Information

**Additional file 1.** In this file we have included three tables detail the composition of the datasets. Table S1 presents the Vir 61 pre-training dataset data distribution. Table S2 shows the Rabies lyssavirus dataset data distribution. Table S3 gives the Flavivirus dataset data distribution.

## Data Availability

The original pre-training dataset Vir61 and the original prediction dataset Flavivirus are available on the figshare (https://figshare.com/s/131bb316a196c4674207), and all experimental data and source code are available on the GitHub via the following link: https://github.com/mingzhaoyan/hostnet.

## Abbreviations

K2V: Vector representation of the K-mers
ASW: Adaptive sliding window
RNN: Recurrent neural networks
GRU: Gated recurrent units
LSTM: Long short-term memory
ENA: European Nucleotide Archive
NCBI: National Center for Biotechnology Information
RC: Repeat and cut
CNN: Convolutional neural networks
BiGRU: Bidirectional gated recurrent unit
BiLSTM: Bidirectional long-short-term-memory
