# Supplementary material for: HostNet: improved sequence representation in deep neural networks for virus-host prediction
Source: BMC Bioinformatics. 2023 Dec 1;24:455. doi: 10.1186/s12859-023-05582-9 (PMC10691023; doi:10.1186/s12859-023-05582-9)
Supplement: Supplementary file 1 — Additional file 1. In this file we have included three tables detail the composition of the datasets. Table S1 presents the Vir 61 pre-training dataset data distribution. Table S2 shows the Rabies lyssavirus dataset data distribution. Table S3 gives the Flavivirus dataset data distribution. [file 12859_2023_5582_MOESM1_ESM.pdf]

Table S1 Vir-61 pre-training dataset data distribution.

Virus type	Counts of genome sequences	Longest genome sequence bps	Shortest genome sequence bps	Counts of virus subtype
Rhabdoviridae	1773	15867	80	218
Mesoniviridae	110	20795	86	16
Togaviridae	10212	22874	72	36
Parvoviridae	71	6334	289	22
Reoviridae	9774	4535	97	70
Phenuiviridae	6217	9549	60	176
Peribunyaviridae	5018	12268	77	270
Nairoviridae	4773	14854	165	76
Nodaviridae	15	3204	1305	5
Asfarviridae	6794	193886	84	2
Poxviridae	45	307691	230	11
Flaviviridae	56548	11520	35	112
Nyamiviridae	17	11840	4708	13
Picornaviridae	2	7537	7537	2
Orthomyxoviridae	246	2421	273	14
Papillomaviridae	2	7428	7428	1
Baculoviridae	340	178733	20	78
Carmotetraviridae	4	6158	6155	1
Mononiviridae	2	41178	41178	1
Nudiviridae	27	231621	96944	11
Permutotetraviridae	4	5698	2482	2
Xinmoviridae	7	13559	6275	7
Dicistroviridae	156	10436	8026	15
Lispiviridae	6	14440	9903	6
Ascoviridae	13	199721	741	4
Alphatetraviridae	12	6625	2448	5
Bidnaviridae	9	6543	6022	1
Medioniviridae	3	25068	20278	2
Chuviridae	52	12004	2291	26
Polydnaviridae	478	140906	263	15
Natareviridae	1	12860	12860	1
Euroniviridae	3	29385	24648	3
Polycipiviridae	16	12155	10315	9
Iridoviridae	16	220222	163023	7
Circoviridae	41	2224	1711	13
Tymoviridae	2	6532	6532	1
Alphasatellitidae	11	1532	1479	2
Birnaviridae	13	6065	2778	4
Metaviridae	1	7510	7510	1
Phasmaviridae	70	7243	1527	13
Genomoviridae	55	2373	2093	34
Hytrosaviridae	5	190291	124279	2
Artoviridae	8	12395	6367	7
Mypoviridae	3	9874	2579	1
Iflaviridae	114	10967	8680	15
Solinviviridae	5	10913	10411	2
Bacilladnaviridae	6	4742	4576	3
Cruliviridae	6	6691	799	2
Sarthroviridae	8	872	502	1
Myriaviridae	1	13517	13517	1
Roniviridae	9	26769	26253	2
Aliusviridae	2	13741	13732	2
Smacoviridae	2	2546	2546	1
Tobaniviridae	1	25960	25960	1
Sinhaliviridae	3	5911	5898	2
Anelloviridae	1	2782	2782	1
Nimaviridae	57	309286	19	1
Wupedeviridae	4	11563	1942	1
Qinviridae	16	6585	1601	8
Yueviridae	4	6858	1212	2
NAV	252	12184	209	16

Table S2 Rabies lyssavirus dataset data distribution.

Host type	Counts of genome sequences	Longest genome sequence bps	Shortest genome sequence bps
Canis lupus	5121	11927	158
Equus caballus	118	11924	163
Bos taurus	2280	11939	117
Tadarida brasiliensis	245	11923	264
Desmodus rotundus	164	11923	164
Artibeus lituratus	90	11922	320
Eptesicus fuscus	460	11924	264
Procyon lotor	558	11939	291
Mephitis mephitis	672	11929	220
Vulpes lagopus	178	11926	211
Lasiurus borealis	96	11924	264
Capra hircus	100	11923	117
Cerdocyon thous	82	11881	å
Nyctereutes procyonoides	101	11928	211
Felis catus	259	11939	157
Vulpes vulpes	683	11939	198
Homo sapiens	478	11928	165

Table S3 Flavivirus dataset data distribution.

Host type	Counts of genome sequences	Longest genome sequence bps	Shortest genome sequence bps
Culex	2424	10380	8025
Ixodes	373	10269	10149
Aedes	6829	10986	9939
